# Supplementary material for: Single-molecule theory of enzymatic inhibition
Source: Nat Commun. 2018 Feb 22;9:779. doi: 10.1038/s41467-018-02995-6 (PMC5823943; doi:10.1038/s41467-018-02995-6)
Supplement: Supplementary file 1 — Supplementary Information [file 41467_2018_2995_MOESM1_ESM.pdf]

Supplementary Figures

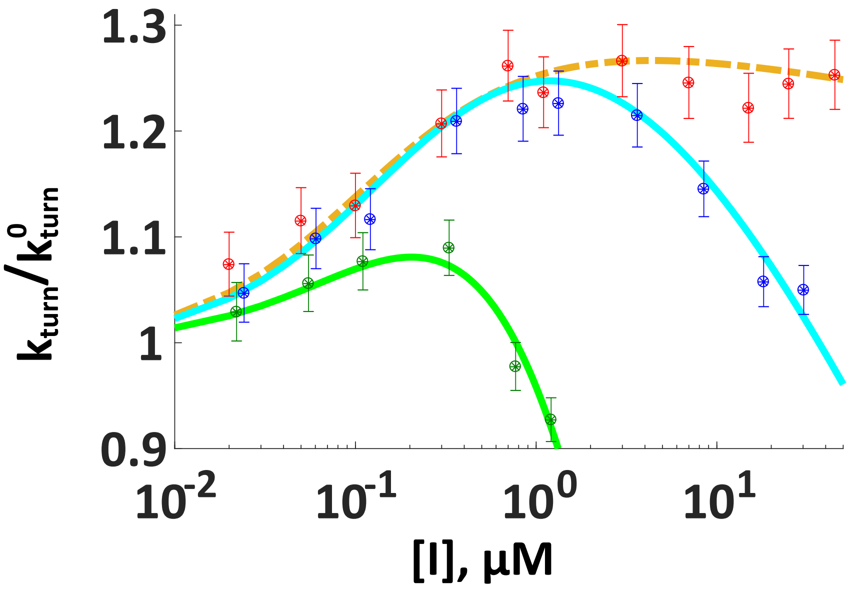

Supplementary Figure 1: Numeric simulations validating results shown in Fig. 5D. Numerical simulations (points and error bars) were preformed to validate the analytical results presented in Fig. 5D (curves). Complete turnover cycles were simulated, with the catalysis time T_{cat} randomly drawn from Log-normal (dashed green), Weibull (solid blue) and Gamma (dash-dot orange) distributions — all with a mean of $7.544\,[ms]$ and variance of $897.85\,\left[ms^2\right]$. All other transition times T_{on}, T_{off}, T^{ESI}_{on} and T^{ESI}_{off} were randomly drawn from exponential distributions with rate constants $k_{on} = 0.1[\mu M^{-1} ms^{-1}]$, $k_{off} = 0.1[ms^{-1}]$, $k^{ESI}_{on} = 2[\mu M^{-1} ms^{-1}]$ and $k^{ESI}_{off} = 1\left[ms^{-1}\right]$, respectively; and the substrate concentration was taken to be $1\,[\mu M]$. Each point in the plot represents an average taken over $N = 5,000$ independent realization. Errors bars were estimated from the sample and represent a spread of ± 2 standard deviations around the sample average.

Supplementary Methods

1 Derivation of Eq. (5) in the main text — Turnover rate for mixed inhibition with generally distributed transition times

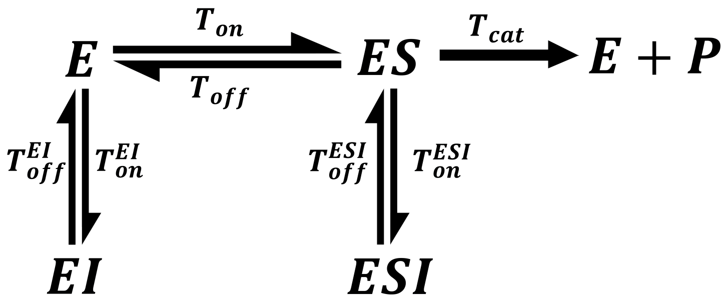

Supplementary Figure 2: A generic scheme for mixed inhibition at the single enzyme level. Transition rates were replaced by generally distributed transition times.

A generic scheme describing enzymatic catalysis under mixed inhibition is illustrated in Fig. S2. We will now analyze this scheme to derive an expression for the enzymatic turnover rate, and will later on show that results in the main text follow as special cases.

Consider a single enzyme that is found initially in its free state (E), and further consider the *random* time it takes the enzyme to reach state ($E + P$). We refer to this time as the turnover time of the reaction, denote it by T_{turn}, and further note that it is given by

$$T_{turn} \equiv F_E = W_E + \begin{cases} F_{ES} & \text{if } T_{on} < T_{on}^{EI} \\ \\ F_{EI} & \text{if } T_{on} > T_{on}^{EI} \, . \end{cases} \tag{1}$$

Here, F_E, F_{ES}, and F_{EI} denote the random times taken to reach the ($E + P$) state, for the first time, having started at states (E), (ES), and (EI) correspondingly. In addition, T_{on} is the random time taken to bind a substrate molecule, T_{on}^{EI} is the random time taken to bind an inhibitor molecule, and $W_E = min(T_{on}, T_{on}^{EI})$ is the random time spent at state (E) prior to its departure. What determines the nature of the transition from state (E) to one of its two neighboring states is whether binding of a substrate molecule preceded that of an inhibitor molecule, $T_{on} < T_{on}^{EI}$, or vice versa $T_{on} > T_{on}^{EI}$. In the former case $T_{turn} = W_E + F_{ES}$ while in the latter we have $T_{turn} = W_E + F_{EI}$. We thus see that T_{turn} cannot be determined in isolation as it also requires knowledge of F_{ES} and F_{EI}.

To proceed, let us first note that F_{EI} is related to other times in the problem via the following relation

$$F_{EI} = W_{EI} + T_{turn} = T_{off}^{EI} + T_{turn} \, , \tag{2}$$

where we noted that $W_{EI} = T_{off}^{EI}$ since the random time spent in state EI prior to its departure is simply the time it takes for the inhibitor to unbind the enzyme. In addition, we see that

$$F_{ES} = W_{ES} + \begin{cases} 0 & \text{if } T_{cat} < T_{off}\,,\, T_{on}^{ESI} \\ \\ T_{turn} & \text{if } T_{off} < T_{cat}\,,\, T_{on}^{ESI} \\ \\ F_{ESI} & \text{if } T_{on}^{ESI} < T_{off}\,,\, T_{cat} \end{cases} \tag{3}$$

where T_{cat} is the random time taken to complete the catalytic step, and T_{off} and T_{on}^{ESI} are respectively the random times taken for the substrate to unbind and the inhibitor to bind the (ES) state. Finally, we note that

$$F_{ESI} = W_{ESI} + F_{ES} = T_{off}^{ESI} + F_{ES}\,, \tag{4}$$

where we have again noted that $W_{ESI} = T_{off}^{ESI}$ since the random time spent in state ESI is simply the time taken for the inhibitor to unbind this state.

Equations (1-4) completely specify the set of relations between the random times T_{turn}, F_{EI}, F_{ES} and F_{ESI}. Taking expectations we then have a set of four equations

$$\begin{gathered} \langle T_{turn}\rangle = \langle W_E\rangle + Pr\left(T_{on} < T_{on}^{EI}\right)\langle F_{ES}\rangle + Pr\left(T_{on} > T_{on}^{EI}\right)\langle F_{EI}\rangle\;, \\ \langle F_{EI}\rangle = \left\langle T_{off}^{EI}\right\rangle + \langle T_{turn}\rangle\;, \\ \langle F_{ES}\rangle = \langle W_{ES}\rangle + Pr\left(T_{off} < T_{cat}\,,\,T_{on}^{ESI}\right)\langle T_{turn}\rangle + Pr\left(T_{on}^{ESI} < T_{off}\,,\,T_{cat}\right)\langle F_{ESI}\rangle\;, \\ \langle F_{ESI}\rangle = \left\langle T_{off}^{ESI}\right\rangle + \langle F_{ES}\rangle\;, \end{gathered} \tag{5}$$

for the unknowns $\langle T_{turn}\rangle\,,\langle F_{EI}\rangle\,,\langle F_{ES}\rangle$ and $\langle F_{ESI}\rangle$, and solving for $\langle T_{turn}\rangle$ gives

$$\langle T_{turn}\rangle = \begin{cases} \frac{\left(1-Pr\left(T_{on}^{ESI}<T_{off},T_{cat}\right)\right)\langle W_E\rangle}{Pr(T_{cat}<T_{off},T_{on}^{ESI})Pr(T_{on}<T_{on}^{EI})} + \frac{\langle W_{ES}\rangle}{Pr(T_{cat}<T_{off},T_{on}^{ESI})} \\ \\ +\frac{\left(1-Pr\left(T_{on}<T_{on}^{EI}\right)\right)\left(1-Pr\left(T_{on}^{ESI}<T_{off},T_{cat}\right)\right)\left\langle T_{off}^{EI}\right\rangle}{Pr(T_{cat}<T_{off},T_{on}^{ESI})Pr(T_{on}<T_{on}^{EI})} \\ \\ +\frac{Pr\left(T_{on}^{ESI}<T_{off},T_{cat}\right)\left\langle T_{off}^{ESI}\right\rangle}{Pr(T_{cat}<T_{off},T_{on}^{ESI})}\,. \end{cases} \tag{6}$$

The right hand side of Eq. (6) depends on certain probabilities and expectations values but these can all be computed given information on the underlying transitions times which govern the problem. Indeed, with $f_X(t)$ denoting the probability density of a random variable X and $\bar{F}_X(t) = Pr(X > t) = 1 - \int_0^t f_X(t')dt'$ as the complimentary cumulative distribution function of this random variable, we have

$$\begin{gathered} Pr\left(T_{on}^{ESI} < T_{off}, T_{cat}\right) = \int_0^\infty f_{T_{on}^{ESI}}(t)\,\bar{F}_{T_{off}}(t)\,\bar{F}_{T_{cat}}(t)\,dt\,, \\ Pr\left(T_{cat} < T_{off}, T_{on}^{ESI}\right) = \int_0^\infty f_{T_{cat}}(t)\,\bar{F}_{T_{off}}(t)\,\bar{F}_{T_{on}^{ESI}}(t)\,dt\,, \\ Pr\left(T_{on} < T_{on}^{EI}\right) = \int_0^\infty f_{T_{on}}(t)\,\bar{F}_{T_{on}^{EI}}(t)\,dt\,. \end{gathered} \tag{7}$$

The mean times $\langle W_E\rangle$ and $\langle W_{ES}\rangle$ can also be written in a similar way exploiting the fact that the time spent at a state is a minimum over the occurrence times of competing processes governing the departure from this state. For example, W_E is nothing but a minimum over T_{on} and T_{on}^{EI}. We thus have $\bar{F}_{W_E}(t) = Pr(W_E > t) = \bar{F}_{T_{on}}(t)\,\bar{F}_{T_{on}^{EI}}(t)$ and hence

$$\langle W_E\rangle = \int_0^\infty \bar{F}_{W_E}(t)dt = \int_0^\infty \bar{F}_{T_{on}}(t)\,\bar{F}_{T_{on}^{EI}}(t)\,dt\,. \tag{8}$$

Similarly, we find that

$$\langle W_{ES} \rangle = \int_0^\infty \bar{F}_{W_{ES}}(t)dt = \int_0^\infty \bar{F}_{T_{off}}(t)\, \bar{F}_{T_{on}^{ESI}}(t)\, \bar{F}_{T_{cat}}(t)dt\,. \tag{9}$$

1.1 Simplified expressions for the case of exponential binding times

The result in Eq. (6) can be simplified by taking advantage of the fact that many substrate molecules independently compete for the binding of the same enzyme. And so, while the stochastic time characterizing the binding of a single substrate molecule may be complex, the amalgamation of many independent binding attempts will follow Poisson statistics. This in turn means that the binding time T_{on} comes from an exponential distribution with density

$$f_{T_{on}}(t) = k_{on}\,[S]\, e^{-k_{on}[S]t}\,. \tag{10}$$

Using the same rational for the binding time of the inhibitor to the enzyme, T_{on}^{EI}, and to the enzyme substrate complex, T_{on}^{ESI}, gives

$$\begin{aligned} f_{T_{on}^{EI}}(t) &= k_{on}^{EI}\,[I]\, e^{-k_{on}^{EI}[I]t}\,, \\ f_{T_{on}^{ESI}}(t) &= k_{on}^{ESI}\,[I]\, e^{-k_{on}^{ESI}[I]t}\,, \end{aligned} \tag{11}$$

for the probability density functions of these random variables.

1.1.1 Turnover in the absence of inhibition

In the absence of inhibitor molecules the probability of binding one is zero, and we thus have $Pr\left(T_{on}^{ESI} < T_{off}, T_{cat}\right) = 0$, $Pr\left(T_{on} < T_{on}^{EI}\right) = 1$, and $Pr\left(T_{cat} < T_{on}^{ESI}, T_{off}\right) = Pr\left(T_{cat} < T_{off}\right)$. It then follows that the mean time spent in state E is simply the time it takes a substrate to bind the enzyme, $\langle W_E \rangle = \langle T_{on} \rangle$, and Eq. (6) becomes

$$\langle T_{turn} \rangle = \frac{\langle T_{on} \rangle + \langle min\left(T_{cat}, T_{off}\right) \rangle}{Pr\left(T_{cat} < T_{off}\right)}\,. \tag{12}$$

Equation (10) then implies that $\langle T_{on} \rangle^{-1} = k_{on}\,[S]$ and rearmament of (12) gives

$$\langle T_{turn} \rangle = \frac{1}{Pr\left(T_{cat} < T_{off}\right) k_{on}\,[S]} + \frac{\langle min\left(T_{cat}, T_{off}\right) \rangle}{Pr\left(T_{cat} < T_{off}\right)}\,. \tag{13}$$

Comparing the result in Eq. (13) to the classical Michaelis–Menten equation $\langle T_{turn} \rangle = \frac{K_m}{v_{max}} \frac{1}{[S]} + \frac{1}{v_{max}}$, we identify the constants

$$\begin{aligned} K_m &= \tfrac{1}{k_{on}\langle min(T_{cat}, T_{off})\rangle} = \tfrac{1}{k_{on}\int_0^\infty \bar{F}_{T_{cat}}(t)\bar{F}_{T_{off}}(t)dt}\,, \\ v_{max} &= \tfrac{Pr(T_{cat} < T_{off})}{\langle min(T_{cat}, T_{off})\rangle} = \tfrac{\int_0^\infty f_{T_{cat}}(t)\bar{F}_{T_{off}}(t)dt}{\int_0^\infty \bar{F}_{T_{cat}}(t)\bar{F}_{T_{off}}(t)dt}\,, \end{aligned} \tag{14}$$

and note that in order to get these expressions we did not make any assumptions on the distributions of the catalysis time T_{cat} and unbinding time T_{off}.

1.1.2 Turnover with inhibition

To progress analysis in the case where inhibitors are present, we first note that Eq. (14) asserts that

$$\int_0^\infty f_{T_{cat}}(t)\, \bar{F}_{T_{off}}(t)\, dt = \frac{v_{max}}{K_m k_{on}}\,, \tag{15}$$

and

$$\int_0^\infty \bar{F}_{T_{cat}}(t)\,\bar{F}_{T_{off}}(t)\,dt = \frac{1}{K_m k_{on}}\,. \tag{16}$$

Using this fact, we define two normalized probability density functions $f_M(t)$ and $f_P(t)$

$$\begin{aligned} f_M(t) &= K_m k_{on} \bar{F}_{T_{cat}}(t)\,\bar{F}_{T_{off}}(t)\ , \\ f_P(t) &= \tfrac{K_m k_{on}}{v_{max}} f_{T_{cat}}(t)\,\bar{F}_{T_{off}}\ , \end{aligned} \tag{17}$$

and their corresponding Laplace transforms $\tilde{f}_M(s)$ and $\tilde{f}_P(s)$

$$\begin{aligned} \tilde{f}_M(s) &= \textstyle\int_0^\infty e^{-st} K_m k_{on} \bar{F}_{T_{cat}}(t)\,\bar{F}_{T_{off}}(t)\,dt\,, \\ \tilde{f}_P(s) &= \textstyle\int_0^\infty e^{-st} \tfrac{K_m k_{on}}{v_{max}} f_{T_{cat}}(t)\,\bar{F}_{T_{off}}(t)dt\,. \end{aligned} \tag{18}$$

Using these definitions, and by use of Eq. (11), Eq. (7) can be simplified and written in the following form

$$\begin{aligned} Pr\left(T_{on}^{ESI} < T_{off}, T_{cat}\right) &= \tfrac{k_{on}^{ESI}[I]}{K_m k_{on}} \textstyle\int_0^\infty e^{-k_{on}^{ESI}[I]t} f_M(t)\,dt = \tfrac{k_{on}^{ESI}[I]}{K_m k_{on}} \tilde{f}_M\left(k_{on}^{ESI}[I]\right)\ , \\ Pr\left(T_{cat} < T_{off}, T_{on}^{ESI}\right) &= \tfrac{v_{max}}{K_m k_{on}} \textstyle\int_0^\infty e^{-k_{on}^{ESI}[I]t} f_P(t)\,dt = \tfrac{v_{max}}{K_m k_{on}} \tilde{f}_P\left(k_{on}^{ESI}[I]\right)\ , \\ Pr\left(T_{on} < T_{on}^{EI}\right) &= \tfrac{k_{on}[S]}{k_{on}[S] + k_{on}^{EI}[I]}\,. \end{aligned} \tag{19}$$

Doing the same for Eqs. (8-9) we find

$$\langle W_E \rangle = \frac{1}{k_{on}[S] + k_{on}^{EI}[I]}\,, \tag{20}$$

$$\langle W_{ES} \rangle = \frac{1}{K_m k_{on}} \int_0^\infty e^{-k_{on}^{ESI}[I]t} f_M(t)\,dt = \frac{1}{K_m k_{on}} \tilde{f}_M\left(k_{on}^{ESI}[I]\right)\ . \tag{21}$$

Substituting Eq. (19-21) back into Eq. (6) the latter can be simplified to give

$$\langle T_{turn} \rangle = \frac{K_m \left(1 + k_{on}^{EI}\left\langle T_{off}^{EI}\right\rangle [I]\right)}{v_{max}} \frac{A([I])}{[S]} + \frac{\left(1 + k_{on}^{ESI}\left\langle T_{off}^{ESI}\right\rangle [I]\right) B([I])}{v_{max}}\,, \tag{22}$$

where

$$A([I]) = \frac{1 - \frac{k_{on}^{ESI}[I]}{K_m k_{on}} \tilde{f}_M\left(k_{on}^{ESI}[I]\right)}{\tilde{f}_P\left(k_{on}^{ESI}[I]\right)}\,, \tag{23}$$

and

$$B([I]) = \frac{\tilde{f}_M\left(k_{on}^{ESI}[I]\right)}{\tilde{f}_P\left(k_{on}^{ESI}[I]\right)}\,. \tag{24}$$

Equations (23-24) coincide with Eqs. (8-9) in the Methods section of the main text. Recalling that $\langle T_{turn} \rangle \equiv 1/k_{turn}$, $K_{EI} = \left(k_{on}^{EI}\left\langle T_{off}^{EI}\right\rangle\right)^{-1}$, and $K_{ESI} = \left(k_{on}^{ESI}\left\langle T_{off}^{ESI}\right\rangle\right)^{-1}$, we see that Eq. (22) coincides with Eq. (5) in the main text, and we will now show that all of the results in the main text could be derived from it.

2 Proof that Eq. (5) in the main text reduces to Eq. (1) when all transition times are exponentially distributed

When all the transition times in Fig. S2 are exponentially distributed Eqs. (10-11) remain valid, and in addition we have

$$
\begin{aligned}
f_{T_{cat}}(t) &= k_{cat} e^{-k_{cat} t}\,, \\
f_{T_{off}}(t) &= k_{off} e^{-k_{off} t}\,, \\
f_{T_{off}^{EI}}(t) &= k_{off}^{EI} e^{-k_{off}^{EI} t}\,, \\
f_{T_{off}^{ESI}}(t) &= k_{off}^{ESI} e^{-k_{off}^{ESI} t}\,,
\end{aligned}
\tag{25}
$$

for the probability density functions of $T_{cat}, T_{off}, T_{off}^{EI}$, and T_{off}^{ESI}. We then find that

$$
Pr\left(T_{cat} < T_{off}\right) = \int_0^\infty f_{T_{cat}}(t)\,\bar{F}_{T_{off}}(t)\,dt = \int_0^\infty k_{cat} e^{-k_{cat} t} e^{-k_{off} t} dt = \frac{k_{cat}}{k_{off} + k_{cat}}\,, \tag{26}
$$

and

$$
\langle min\left(T_{cat}, T_{off}\right)\rangle = \int_0^\infty \bar{F}_{T_{cat}}(t)\,\bar{F}_{T_{off}}(t)\,dt = \int_0^\infty e^{-k_{cat} t} e^{-k_{off} t} dt = \frac{1}{k_{off} + k_{cat}}\,, \tag{27}
$$

from which it follows that

$$
\begin{aligned}
v_{max} &= \tfrac{Pr(T_{cat} < T_{off})}{\langle min(T_{cat}, T_{off})\rangle} = k_{cat}\,, \\
K_m &= \tfrac{1}{k_{on}\langle min(T_{cat}, T_{off})\rangle} = \tfrac{k_{off} + k_{cat}}{k_{on}}\,.
\end{aligned}
\tag{28}
$$

In addition, from Eqs. (17-18) we now have

$$
\begin{aligned}
\tilde{f}_M(k_{on}^{ESI}[I]) &= \tfrac{k_{off} + k_{cat}}{k_{on}} k_{on} \int_0^\infty e^{-k_{on}^{ESI}[I]t} e^{-k_{cat} t} e^{-k_{off} t} dt = \tfrac{k_{off} + k_{cat}}{k_{off} + k_{cat} + k_{on}^{ESI}[I]}\,, \\
\tilde{f}_P(k_{on}^{ESI}[I]) &= \tfrac{\frac{k_{off} + k_{cat}}{k_{on}} k_{on}}{k_{cat}} \int_0^\infty e^{-k_{on}^{ESI}[I]t} k_{cat} e^{-k_{cat} t} e^{-k_{off} t} dt = \tfrac{k_{off} + k_{cat}}{k_{off} + k_{cat} + k_{on}^{ESI}[I]}\,.
\end{aligned}
\tag{29}
$$

Substituting the above back into Eq. (23-24) we conclude that

$$
A\left([I]\right) = \frac{k_{off} + k_{cat} + k_{on}^{ESI}[I]}{k_{off} + k_{cat}} \left[1 - \frac{k_{on}^{ESI}[I]}{k_{off} + k_{cat}} \cdot \frac{k_{off} + k_{cat}}{k_{off} + k_{cat} + k_{on}^{ESI}[I]}\right] = 1\,, \tag{30}
$$

and

$$
B\left([I]\right) = 1\,. \tag{31}
$$

Equation (25) moreover asserts that $k_{off}^{EI} = 1/\left\langle T_{off}^{EI}\right\rangle$ and $k_{off}^{ESI} = 1/\left\langle T_{off}^{ESI}\right\rangle$, which in turn means that

$$
\langle T_{turn}\rangle = \frac{K_m\left(1 + \frac{k_{on}^{EI}}{k_{off}^{EI}}[I]\right)}{v_{max}} \frac{1}{[S]} + \frac{\left(1 + \frac{k_{on}^{ESI}}{k_{off}^{ESI}}[I]\right)}{v_{max}}\,, \tag{32}
$$

and we see that the result in Eq. (32) coincides with Eq. (1) in the main text.

3 Derivation of Eq. (2) in the main text from Eq. (5) — Turnover rate for competitive inhibition with generally distributed transition times

Competitive inhibition can be seen as a special case of mixed inhibition in which the binding rate of the inhibitor to the enzyme-substrate complex is zero ($k_{on}^{ESI} = 0$). Keeping in mind Eqs. (15-17), and the definition in Eq. (18), we see that

$$\begin{aligned} \tilde{f}_M(k_{on}^{ESI}[I]=0) &= \int_0^\infty K_m k_{on} \bar{F}_{T_{cat}}(t)\, \bar{F}_{T_{off}}(t)\, dt = \int_0^\infty f_M(t) dt = 1\,, \\ \tilde{f}_P(k_{on}^{ESI}[I]=0) &= \int_0^\infty \frac{K_m k_{on}}{v_{max}} f_{T_{cat}}(t)\, \bar{F}_{T_{off}}(t) dt = \int_0^\infty f_P(t) dt = 1\,, \end{aligned} \tag{33}$$

and thus have

$$A\left(k_{on}^{ESI}[I]=0\right) = B\left(k_{on}^{ESI}[I]=0\right) = 1\,. \tag{34}$$

Equation (22) then becomes

$$\langle T_{turn} \rangle = \frac{K_m \left(1 + k_{on}^{EI} \left\langle T_{off}^{EI} \right\rangle [I]\right)}{v_{max}} \frac{1}{[S]} + \frac{1}{v_{max}}\,, \tag{35}$$

which coincides with Eq. (2) in the main text.

4 Derivation of Eq. (3) in the main text from Eq. (5) — Turnover rate for uncompetitive inhibition with generally distributed transition times

Uncompetitive inhibition can also be seen as a special case of mixed inhibition. Here, the binding rate of the inhibitor to the free enzyme is zero ($k_{on}^{EI} = 0$), and since $A[I]$ and $B[I]$ do not dependent on k_{on}^{EI}, Eq. (22) reduces to

$$\langle T_{turn} \rangle = \frac{K_m}{v_{max}} \frac{A([I])}{[S]} + \frac{\left(1 + k_{on}^{ESI} \left\langle T_{off}^{ESI} \right\rangle [I]\right) B([I])}{v_{max}}\,, \tag{36}$$

which coincides with Eq. (3) in the main text.

5 The two-state model

The two-state model disscussed in the main text, and illustrated in Fig. S3, is a special case of the general scheme for uncompetitive inhibition (Fig. 4, main text). Indeed, the two can be shown to coincide by allowing the catalysis time in the general scheme to come from a distribution whose density is

$$f_{T_{cat}}(t) = p k_{cat}^{(1)} e^{-k_{cat}^{(1)} t} + (1-p) k_{cat}^{(2)} e^{-k_{cat}^{(2)} t}\,, \tag{37}$$

and otherwise taking all transition times to be exponentially distributed with proper rates. To see this, note that in the two state model the ES complex can be found in one of two states: ES_1 or ES_2. However, these states have the same substrate unbinding rate, k_{off}, and inhibitor binding rate, $k_{on}^{ESI}[I]$, and only differ in their catalytic rates which are correspondingly given by $k_{cat}^{(1)}$ and $k_{cat}^{(2)}$. Moreover, a transition from the free enzyme E occurs with rate $k_{on}[S]$ and leads

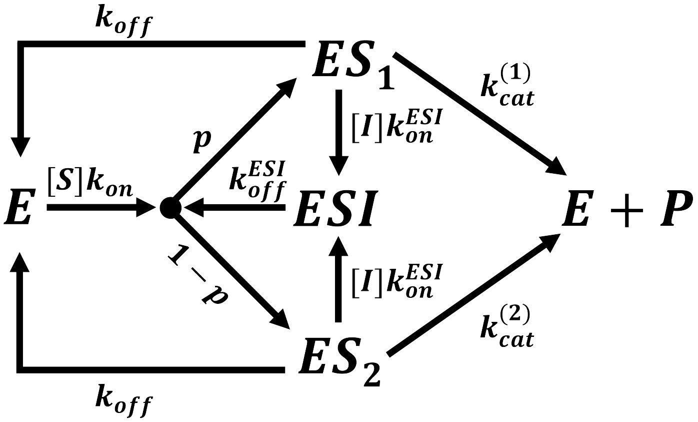

Supplementary Figure 3: A two-state model that is a particular instance of the generic scheme in Fig. 4 (Main Text). Binding of a substrate to the enzyme can occur in one of two ways with probabilities p and $(1-p)$, each leading to a different enzyme substrate complex (ES_1 or ES_2), that is furthermore equipped with a distinct catalytic rate ($k_{cat}^{(1)}$ or $k_{cat}^{(2)}$). The inhibitor binds each of the enzyme-substrate complexes with the same rate, $k_{on}^{ESI}[I]$, and when it unbinds these states are once again reached with probabilities p and $(1-p)$.

to ES_1 with probability p and to ES_2 with probability $1-p$, and these states are reached with the exact same probabilities after an inhibitor unbinds, with rate k_{off}^{ESI}, from the ESI complex. We thus see that ES_1 and ES_2 could be effectively merged into a single ES state whose catalysis rate is randomly drawn to be $k_{cat}^{(1)}$ with probability p and $k_{cat}^{(2)}$ with probability $1-p$ every time this state is visited. This asserts that the probability density function of T_{cat} is given by Eq. (37) above, and we further note that from the construction of the two state model it follows that T_{on},T_{off},T_{on}^{ESI} and T_{off}^{ESI} are all exponentially distributed with rates $k_{on}[S]$, k_{off}, $k_{on}^{ESI}[I]$ and k_{off}^{ESI}, respectively.

5.1 Derivation of explicit expressions for the functions A([I]) and B([I]) in the case of the two-state model

With the above at hand we can derive explicit expressions for $A\left([I]\right)$ and $B\left([I]\right)$. We first note that in the case of the two state model Eq. (18) can be written as

$$\tilde{f}_M(k_{on}^{ESI}\,[I]) = K_m k_{on}\cdot\int_0^\infty e^{-\left(k_{on}^{ESI}[I]+k_{off}\right)t}\bar{F}_{T_{cat}}\left(t\right)dt = K_m k_{on}\cdot\frac{1-\tilde{f}_{T_{cat}}(k_{off}+k_{on}^{ESI}[I])}{k_{off}+k_{on}^{ESI}[I]}\,,$$

$$\tilde{f}_P(k_{on}^{ESI}\,[I]) = \frac{K_m k_{on}}{v_{max}}\int_0^\infty e^{-\left(k_{on}^{ESI}[I]+k_{off}\right)t}f_{T_{cat}}\left(t\right)dt = \frac{K_m k_{on}}{v_{max}}\cdot\tilde{f}_{T_{cat}}(k_{off}+k_{on}^{ESI}\,[I])\,, \tag{38}$$

with

$$\tilde{f}_{T_{cat}}(s) = \int_0^\infty e^{-st}f_{T_{cat}}(t)dt = p\frac{k_{cat}^{(1)}}{k_{cat}^{(1)}+s} + (1-p)\frac{k_{cat}^{(2)}}{k_{cat}^{(2)}+s}\,, \tag{39}$$

standing for the Laplace transform of $f_{T_{cat}}\left(t\right)$. Substituting Eq. (38) into Eqs. (23-24) then gives

$$A\left([I]\right) = \frac{v_{max}}{\left(k_{off}+k_{on}^{ESI}\,[I]\right)K_m k_{on}}\left(\frac{k_{off}}{\tilde{f}_{T_{cat}}(k_{off}+k_{on}^{ESI}\,[I])} + k_{on}^{ESI}\,[I]\right)\,, \tag{40}$$

and

$$B\left([I]\right) = \frac{v_{max}}{\left(k_{off} + k_{on}^{ESI}\left[I\right]\right)} \left(\frac{1}{\tilde{f}_{T_{cat}}\left(k_{off} + k_{on}^{ESI}\left[I\right]\right)} - 1 \right) . \tag{41}$$

To further proceed, we observe that in the case of the two state model Eq. (14) reduces to

$$\begin{aligned} v_{max} &= \frac{\int_0^\infty f_{T_{cat}}(t) e^{-k_{off}t} dt}{\int_0^\infty \bar{F}_{T_{cat}}(t) e^{-k_{off}t} dt} = \frac{k_{off}\tilde{f}_{T_{cat}}(k_{off})}{1-\tilde{f}_{T_{cat}}(k_{off})} = \frac{k_{off}(pk_{cat}^{(1)}+(1-p)k_{cat}^{(2)})+k_{cat}^{(1)}k_{cat}^{(2)}}{(1-p)k_{cat}^{(1)}+pk_{cat}^{(2)}+k_{off}} , \\ K_m &= \frac{1}{k_{on}\int_0^\infty \bar{F}_{T_{cat}}(t) e^{-k_{off}t} dt} = \frac{k_{off}}{k_{on}\left(1-\tilde{f}_{T_{cat}}(k_{off})\right)} = \frac{(k_{cat}^{(1)}+k_{off})(k_{cat}^{(2)}+k_{off})}{k_{on}\left((1-p)k_{cat}^{(1)}+pk_{cat}^{(2)}+k_{off}\right)} . \end{aligned} \tag{42}$$

Substituting Eq. (42) into Eqs. (40-41) and making use of Eq. (39) we find

$$A([I]) = 1 - \frac{k_{off}(1-p)p\left(\frac{1}{k_{cat}^{(1)}} - \frac{1}{k_{cat}^{(2)}}\right)^2 k_{on}^{ESI}\left[I\right]}{\left(1+\frac{k_{off}}{k_{cat}^{(1)}}\right)\left(1+\frac{k_{off}}{k_{cat}^{(2)}}\right)\left(1+\left(\frac{1-p}{k_{cat}^{(1)}}+\frac{p}{k_{cat}^{(2)}}\right)\left(k_{off}+k_{on}^{ESI}\left[I\right]\right)\right)} , \tag{43}$$

and

$$B([I]) = 1 - \frac{(1-p)p\left(\frac{1}{k_{cat}^{(1)}} - \frac{1}{k_{cat}^{(2)}}\right)^2 k_{on}^{ESI}\left[I\right]}{\left(\frac{p}{k_{cat}^{(1)}}+\frac{1-p}{k_{cat}^{(2)}}+\frac{k_{off}}{k_{cat}^{(1)}k_{cat}^{(2)}}\right)\left(1+\left(\frac{1-p}{k_{cat}^{(1)}}+\frac{p}{k_{cat}^{(2)}}\right)\left(k_{off}+k_{on}^{ESI}\left[I\right]\right)\right)} . \tag{44}$$

It could now also be observed that at high inhibitor concentrations both $A([I])$ and $B([I])$ approach asymptotic values

$$A([I] \to \infty) \simeq 1 - \frac{k_{off}(1-p)p\left(\frac{1}{k_{cat}^{(1)}} - \frac{1}{k_{cat}^{(2)}}\right)^2}{\left(1+\frac{k_{off}}{k_{cat}^{(1)}}\right)\left(1+\frac{k_{off}}{k_{cat}^{(2)}}\right)\left(\frac{1-p}{k_{cat}^{(1)}}+\frac{p}{k_{cat}^{(2)}}\right)} , \tag{45}$$

and

$$B([I] \to \infty) \simeq 1 - \frac{(1-p)p\left(\frac{1}{k_{cat}^{(1)}} - \frac{1}{k_{cat}^{(2)}}\right)^2}{\left(\frac{p}{k_{cat}^{(1)}}+\frac{1-p}{k_{cat}^{(2)}}+\frac{k_{off}}{k_{cat}^{(1)}k_{cat}^{(2)}}\right)\left(\frac{1-p}{k_{cat}^{(1)}}+\frac{p}{k_{cat}^{(2)}}\right)} . \tag{46}$$

6 Derivation of a general condition for the emergence of inhibitor-activator duality

To derive a general condition for the emergence of inhibitor-activator duality we start from the expression for the mean turnover time in Eq. (22), and ask when will $\frac{d\langle T_{turn}\rangle}{d[I]}|_{[I]=0} < 0$? In other words, we would like to determine when will an increase in the concentration of the inhibitor, from an initial value of zero, result in a decrease of the mean turnover time, and hence in an increase of the turnover rate. To answer this question we first note that

$$\begin{aligned} \frac{d\langle T_{turn}\rangle}{d[I]}|_{[I]=0} &= \frac{K_m k_{on}^{EI}\left\langle T_{off}^{EI}\right\rangle}{v_{max}[S]} + \frac{k_{on}^{ESI}\left\langle T_{off}^{ESI}\right\rangle}{v_{max}} \\ &+ \frac{K_m}{v_{max}[S]}\frac{dA([I])}{d[I]}|_{[I]=0} + \frac{1}{v_{max}}\frac{dB([I])}{d[I]}|_{[I]=0} . \end{aligned} \tag{47}$$

Now, since $\tilde{f}_M(s)$ and $\tilde{f}_P(s)$ are the Laplace transforms of the random variables M and P defined by the normalized densities in Eq. (17) we have

$$\tilde{f}_M\left(k_{on}^{ESI}[I]\right) = 1 - k_{on}^{ESI}[I]\langle M\rangle + O([I]^2)\,, \tag{48}$$

and

$$\tilde{f}_P\left(k_{on}^{ESI}[I]\right) = 1 - k_{on}^{ESI}[I]\langle P\rangle + O([I]^2)\,, \tag{49}$$

from which we find that

$$A([I]) = \frac{1 - \frac{k_{on}^{ESI}[I]}{K_m k_{on}}\tilde{f}_M\left(k_{on}^{ESI}[I]\right)}{\tilde{f}_P\left(k_{on}^{ESI}[I]\right)} = 1 + k_{on}^{ESI}[I]\langle P\rangle - \frac{k_{on}^{ESI}[I]}{K_m k_{on}} + O([I]^2)\,, \tag{50}$$

and

$$B([I]) = \frac{\tilde{f}_M\left(k_{on}^{ESI}[I]\right)}{\tilde{f}_P\left(k_{on}^{ESI}[I]\right)} = 1 + k_{on}^{ESI}[I]\langle P\rangle - k_{on}^{ESI}[I]\langle M\rangle + O([I]^2)\,. \tag{51}$$

Plugging these equations back into Eq. (47) we find

$$\begin{aligned} &\tfrac{d\langle T_{turn}\rangle}{d[I]}|_{[I]=0} = \tfrac{K_m k_{on}^{EI}\left\langle T_{off}^{EI}\right\rangle}{v_{max}[S]} + \tfrac{k_{on}^{ESI}\left\langle T_{off}^{ESI}\right\rangle}{v_{max}} \\ &\tfrac{K_m\left(k_{on}^{ESI}\langle P\rangle - \frac{k_{on}^{ESI}}{K_m k_{on}}\right)}{v_{max}[S]} + \tfrac{k_{on}^{ESI}\langle P\rangle - k_{on}^{ESI}\langle M\rangle}{v_{max}}\,. \end{aligned} \tag{52}$$

Rearranging we see that $\frac{d\langle T_{turn}\rangle}{d[I]}|_{[I]=0} < 0$ if and only if

$$\frac{K_m k_{on}^{EI}\left\langle T_{off}^{EI}\right\rangle}{k_{on}^{ESI}[S]} + \left(1 + \frac{K_m}{[S]}\right)\langle P\rangle + \left\langle T_{off}^{ESI}\right\rangle - \frac{1}{k_{on}[S]} < \langle M\rangle\,. \tag{53}$$

To make sense of this condition, and some more progress, we return to the definitions of $\langle P\rangle$ and $\langle M\rangle$ and recall that these are given by

$$\langle M\rangle = K_m k_{on}\int_0^\infty t\cdot \bar{F}_{T_{cat}}(t)\,\bar{F}_{T_{off}}(t)\,dt\,, \tag{54}$$

and

$$\langle P\rangle = \frac{K_m k_{on}}{v_{max}}\int_0^\infty t\cdot f_{T_{cat}}(t)\,\bar{F}_{T_{off}}(t)dt\,, \tag{55}$$

which in turn means that they could be related to the life time, $W_{ES}^0 = min\left(T_{cat}, T_{off}\right)$, of the enzyme substrate complex in the absence of inhibition. Indeed, recalling the definitions of K_m and v_{max} in Eq. (14) we observe that

$$\begin{aligned} &f_P(t) = \tfrac{K_m k_{on}}{v_{max}} f_{T_{cat}}(t)\,\bar{F}_{T_{off}}(t) = \tfrac{f_{T_{cat}}(t)\bar{F}_{T_{off}}(t)}{Pr(T_{cat}<T_{off})} \\ &= f_{\{T_{cat}|T_{cat}<T_{off}\}}(t) = f_{\{W_{ES}^0|ES\to E+P\}}(t)\,, \end{aligned} \tag{56}$$

where $f_{\{W_{ES}^0|ES\to E+P\}}(t)$ is simply the probability density function of the life time W_{ES}^0 given that the stay in the ES state resulted in product formation (catalysis occurred prior to unbinding). This in turn means that

$$\langle P\rangle = \int_0^\infty t\cdot f_{\{W_{ES}^0|ES\to E+P\}}(t)dt = \left\langle W_{ES}^0|ES\to E+P\right\rangle\,. \tag{57}$$

Regarding $\langle M\rangle$, we first note that

$$\begin{aligned}\bar{F}_{W_{ES}^0}(t) &= Pr\left(W_{ES}^0 > t\right) = Pr\left(min\left(T_{cat}, T_{off}\right) > t\right) \\ &= Pr\left(T_{cat} > t\right) Pr\left(T_{off} > t\right) = \bar{F}_{T_{cat}}(t)\, \bar{F}_{T_{off}}(t)\ ,\end{aligned} \tag{58}$$

i.e., that the life time of the ES state (in the absence of inhibition) is larger than t if and only if neither catalysis nor unbinding occurred by that time. Equation (54) could then be written as

$$\langle M\rangle = K_m k_{on} \int_0^\infty t\cdot \bar{F}_{T_{cat}}(t)\, \bar{F}_{T_{off}}(t)\, dt = \frac{\int_0^\infty t\cdot \bar{F}_{T_{cat}}(t)\, \bar{F}_{T_{off}}(t)\, dt}{\langle min\left(T_{cat}, T_{off}\right)\rangle} = \frac{\int_0^\infty t\cdot \bar{F}_{W_{ES}^0}(t)\, dt}{\langle W_{ES}^0\rangle}\ , \tag{59}$$

where we have once again used the definition of K_m in Eq. (14). The nominator can be worked out using integration by parts and we find

$$\int_0^\infty t\cdot \bar{F}_{W_{ES}^0}(t)\, dt = \frac{t^2}{2}\bar{F}_{W_{ES}^0}(t)\bigg|_0^\infty + \frac{1}{2}\int_0^\infty t^2\cdot f_{W_{ES}^0}(t)dt = \lim_{t\to\infty}\left\{\frac{1}{2}t^2\bar{F}_{W_{ES}^0}(t)\right\} + \frac{1}{2}\left\langle \left(W_{ES}^0\right)^2\right\rangle\ . \tag{60}$$

We now assume that $f_{W_{ES}^0}(t)$ decays to zero "fast enough" in the sense that there exists some $\varepsilon > 0$ such that $\lim_{t\to\infty}\left\{f_{W_{ES}^0}(t)/t^{-(3+\varepsilon)}\right\} = 0$. This condition asserts that $\lim_{t\to\infty}\left\{\frac{1}{2}t^2\bar{F}_{W_{ES}^0}(t)\right\} = \lim_{t\to\infty}\left\{\frac{1}{2}t^2\int_t^\infty f_{W_{ES}^0}(z)dz\right\} = 0$ and that $\frac{1}{2}\int_0^\infty t^2\cdot f_{W_{ES}^0}(t)dt = \frac{1}{2}\left\langle\left(W_{ES}^0\right)^2\right\rangle < \infty$. Substituting back into Eq. (59) we obtain

$$\langle M\rangle = \frac{1}{2}\frac{\left\langle\left(W_{ES}^0\right)^2\right\rangle}{\langle W_{ES}^0\rangle} = \frac{1}{2}\langle W_{ES}^0\rangle\left(CV_{W_{ES}^0}^2 + 1\right)\ , \tag{61}$$

where $CV_{W_{ES}^0}^2 \equiv \frac{\sigma^2\left(W_{ES}^0\right)}{\langle W_{ES}^0\rangle^2} = \frac{\left\langle\left(W_{ES}^0\right)^2\right\rangle - \langle W_{ES}^0\rangle^2}{\langle W_{ES}^0\rangle^2}$ is the normalized variance, a.k.a coefficient of variation, of W_{ES}^0.

Substituting Eqs. (57) and (61) back into Eq. (53) we rewrite the condition for the emergence of inhibitor-activator duality in terms of the mean, conditional mean, and coefficient of variation in the stochastic life time of the ES complex in the absence of inhibition

$$\begin{aligned}&\tfrac{1}{2}\langle W_{ES}^0\rangle\left(CV_{W_{ES}^0}^2 + 1\right) > \\ &\tfrac{k_{on}^{EI}K_m\langle T_{off}^{EI}\rangle}{k_{on}^{ESI}[S]} + \left(1 + \tfrac{K_m}{[S]}\right)\langle W_{ES}^0 | ES \to E + P\rangle + \left\langle T_{off}^{ESI}\right\rangle - \tfrac{1}{k_{on}[S]}\ .\end{aligned} \tag{62}$$

Rearranging and recalling that $K_m k_{on} = \langle W_{ES}^0\rangle^{-1}$ we get

$$\frac{\langle T_{mix}\rangle + \left\langle T_{off}^{ESI}\right\rangle}{\langle W_{ES}^0\rangle} < \frac{1}{2}\left(CV_{W_{ES}^0}^2 - 1\right) + \left(1 - \frac{\langle W_{ES}^0 | ES \to E + P\rangle}{\langle W_{ES}^0\rangle}\right)\left(1 + \frac{K_m}{[S]}\right)\ , \tag{63}$$

where $\langle T_{mix}\rangle \equiv \frac{k_{on}^{EI}K_m}{k_{on}^{ESI}[S]}\left\langle T_{off}^{EI}\right\rangle$. Equation (63) provides a general condition for the emergence of inhibitor activator duality.

6.1 Derivation of Eq. (11) in the Methods section

Equation (11) in the Methods section of the main text can be derived from Eq. (63) by noting that in the case of uncompetitive inhibition $k_{on}^{EI} = 0$ and $\langle T_{mix} \rangle = 0$. Equation (63) then reduces to

$$\frac{\left\langle T_{off}^{ESI} \right\rangle}{\langle W_{ES}^0 \rangle} < \frac{1}{2}\left(CV_{W_{ES}^0}^2 - 1\right) + \left(1 - \frac{\langle W_{ES}^0 | ES \to E + P \rangle}{\langle W_{ES}^0 \rangle}\right)\left(1 + \frac{K_m}{[S]}\right), \qquad (64)$$

which coincides with Eq. (11) in the Methods section of the main text.

6.2 Derivation of Eq. (4) in the main text

To derive Eq. (4) in the main text from Eq. (11) in the Methods (Eq. (64) above), we first recall that $K_m k_{on} = \left\langle W_{ES}^0 \right\rangle^{-1}$ and slightly rearrange Eq. (64) to give

$$\langle T_{off}^{ESI} \rangle < \frac{1}{2K_m k_{on}}\left(CV_{W_{ES}^0}^2 - 1\right) + \left(\frac{1}{K_m k_{on}} - \langle W_{ES}^0 | ES \to E + P \rangle\right)\left(1 + \frac{K_m}{[S]}\right). \qquad (65)$$

We will now show that when substrate unbinding times are taken from the exponential distribution $\left\langle W_{ES}^0 | ES \to E + P \right\rangle$ could be expressed in terms of $CV_{W_{ES}^0}^2$ to greatly simplify the above condition.

We start by noting that Eqs. (59) and (61) could be used to give

$$\frac{1}{2K_m k_{on}}\left(CV_{W_{ES}^0}^2 + 1\right) = K_m k_{on} \int_0^\infty t \cdot \bar{F}_{T_{cat}}(t)\, \bar{F}_{T_{off}}(t)\, dt. \qquad (66)$$

However, when unbinding times are taken from the exponential distribution, $\bar{F}_{T_{off}}(t) = e^{-k_{off} \cdot t}$, and the integral on the right hand side of Eq. (66) reduces to

$$\int_0^\infty t \cdot \bar{F}_{T_{cat}}(t)\, \bar{F}_{T_{off}}(t)\, dt = \int_0^\infty t \cdot \bar{F}_{T_{cat}}(t)\, e^{-k_{off} \cdot t} dt = -\frac{\partial}{\partial k_{off}} \int_0^\infty \bar{F}_{T_{cat}}(t)\, e^{-k_{off} \cdot t} dt. \qquad (67)$$

Integrating by parts, we find

$$\int_0^\infty t \cdot \bar{F}_{T_{cat}}(t)\, e^{-k_{off} \cdot t} dt = -\frac{\partial}{\partial k_{off}}\left[\frac{1 - \tilde{f}_{T_{cat}}(k_{off})}{k_{off}}\right] = \frac{1 - \tilde{f}_{T_{cat}}(k_{off}) + k_{off} \cdot \tilde{f'}_{T_{cat}}(k_{off})}{k_{off}^2}, \qquad (68)$$

where $\tilde{f}'_{T_{cat}}(k_{off})$ is the derivative, with respect to k_{off}, of the Laplace transform of $f_{T_{cat}}(t)$. Substituting Eq. (68) back into Eq. (66), we conclude that

$$\frac{1}{2K_m k_{on}}\left(CV_{W_{ES}^0}^2 + 1\right) = \frac{K_m k_{on}}{k_{off}^2}\left[1 - \tilde{f}_{T_{cat}}(k_{off}) + k_{off} \cdot \tilde{f'}_{T_{cat}}(k_{off})\right], \qquad (69)$$

and solving Eq. (69) for $\tilde{f}'_{T_{cat}}(k_{off})$ then gives

$$\tilde{f'}_{T_{cat}}(k_{off}) = \frac{k_{off}}{2\left(K_m k_{on}\right)^2}\left(CV_{W_{ES}^0}^2 + 1\right) + \frac{\tilde{f}_{T_{cat}}(k_{off}) - 1}{k_{off}}. \qquad (70)$$

We now go back to treat $\left\langle W_{ES}^0 | ES \to E + P \right\rangle$. Combining Eqs. (56) & (57) we obtain

$$\left\langle W_{ES}^0 | ES \to E + P \right\rangle = \frac{K_m k_{on} \int_0^\infty t \cdot f_{T_{cat}}(t)\, \bar{F}_{T_{off}}(t) dt}{v_{max}}. \qquad (71)$$

The integral on the right hand side could once again be treated by taking advantage of the fact that $\bar{F}_{T_{off}}(t) = e^{-k_{off}\cdot t}$. Doing so, we find

$$\int_0^\infty t \cdot f_{T_{cat}}(t)\,\bar{F}_{T_{off}}(t)dt = -\frac{\partial}{\partial k_{off}}\int_0^\infty f_{T_{cat}}(t)\,e^{-k_{off}\cdot t}dt = -\tilde{f}'_{T_{cat}}(k_{off})\,, \tag{72}$$

and substituting back into Eq. (71) gives

$$\left\langle W_{ES}^0 | ES \to E + P \right\rangle = -\frac{K_m k_{on} \tilde{f}'_{T_{cat}}(k_{off})}{v_{max}}\,. \tag{73}$$

Substituting Eq. (70) into Eq. (73) then gives

$$\left\langle W_{ES}^0 | ES \to E + P \right\rangle = -\frac{K_m k_{on}}{v_{max}}\left[\frac{k_{off}}{2\left(K_m k_{on}\right)^2}\left(CV_{W_{ES}^0}^2 + 1\right) + \frac{\tilde{f}_{T_{cat}}(k_{off}) - 1}{k_{off}}\right]. \tag{74}$$

Finally, we use Eq. (15) to obtain

$$\frac{v_{max}}{K_m k_{on}} = \int_0^\infty f_{T_{cat}}(t)\,\bar{F}_{T_{off}}(t)\,dt = \int_0^\infty f_{T_{cat}}(t)\,e^{-k_{off}\cdot t}dt = \tilde{f}_{T_{cat}}(k_{off})\,, \tag{75}$$

and substituting for $\tilde{f}_{T_{cat}}(k_{off})$ in Eq. (74) then gives

$$\left\langle W_{ES}^0 | ES \to E + P \right\rangle = -\frac{k_{off}}{2K_m k_{on} v_{max}}\left(CV_{W_{ES}^0}^2 + 1\right) - \frac{v_{max} - K_m k_{on}}{k_{off} v_{max}}\,. \tag{76}$$

To finish, we substitute Eq. (76) back into Eq. (65) to obtain

$$\begin{aligned} &\left\langle T_{off}^{ESI} \right\rangle < \tfrac{1}{2K_m k_{on}}\left(CV_{W_{ES}^0}^2 - 1\right) \\ &\left(\tfrac{1}{K_m k_{on}} + \tfrac{k_{off}}{2K_m k_{on} v_{max}}\left(CV_{W_{ES}^0}^2 + 1\right) + \tfrac{v_{max} - K_m k_{on}}{k_{off} v_{max}}\right)\left(1 + \tfrac{K_m}{[S]}\right), \end{aligned} \tag{77}$$

and further rearrangement of Eq. (77) gives

$$\begin{aligned} \left\langle T_{off}^{ESI} \right\rangle &< \tfrac{1}{v_{max}}\left[\tfrac{v_{max} + k_{off}}{K_m k_{on}} + \tfrac{k_{off}}{k_{on}[S]}\right]\tfrac{1}{2}\left(CV_{W_{ES}^0}^2 - 1\right) \\ &+ \tfrac{1}{v_{max}}\left(\tfrac{v_{max} + k_{off}}{K_m k_{on}} - \tfrac{K_m k_{on} - v_{max}}{k_{off}}\right)\left(1 + \tfrac{K_m}{[S]}\right). \end{aligned} \tag{78}$$

We now note that in the case of exponential substrate unbinding times, Eq. (14) becomes

$$\begin{aligned} K_m &= \tfrac{1}{k_{on}\int_0^\infty \bar{F}_{T_{cat}}(t) e^{-k_{off}\cdot t}dt} = \tfrac{1}{k_{on}}\tfrac{k_{off}}{1 - \tilde{f}_{T_{cat}}(k_{off})} \\ v_{max} &= \tfrac{\int_0^\infty f_{T_{cat}}(t) e^{-k_{off}\cdot t}dt}{\int_0^\infty \bar{F}_{T_{cat}}(t) e^{-k_{off}\cdot t}dt} = \tfrac{k_{off}\tilde{f}_{T_{cat}}(k_{off})}{1 - \tilde{f}_{T_{cat}}(k_{off})}\,, \end{aligned} \tag{79}$$

and it is then easy to see that in this case

$$K_m k_{on} = k_{off} + v_{max}\,. \tag{80}$$

Substituting Eq. (80) into Eq. (78) kills the second term in this equation and simplifies the first to give

$$\left\langle T_{off}^{ESI} \right\rangle < \frac{1}{2}\left(CV_{W_{ES}^0}^2 - 1\right)\frac{1}{v_{max}}\left[1 + \frac{k_{off}}{k_{on}[S]}\right]. \tag{81}$$

Equation (81) coincides with Eq. (4) in the main text.
